# Understanding Mobile OTT Service Users’ Resistance to Participation in Wireless D2D Caching Networks

**DOI:** 10.3390/bs14030158

**Published:** 2024-02-21

**Authors:** Yumi Jang, Seongcheol Kim

**Affiliations:** 1 Smart Media Service Research Center, Korea University, Seoul 02841, Republic of Korea; pamp999@korea.ac.kr; 2 School of Media and Communication, Korea University, Seoul 02841, Republic of Korea

**Keywords:** mobile OTT service, wireless D2D caching networks, perceived cost, user resistance, participation intention

## Abstract

With the explosive pace of mobile over-the-top (OTT) video content streaming services, mobile network traffic has seen unprecedented growth in recent years. However, the limitation of antenna performance, the burden of investment cost, and restricted resources hinder improving the current mobile networks’ functionality. Accordingly, wireless device-to-device (D2D) caching networks came to the fore as one of the competitive alternatives for alleviating the overloads of mobile network traffic. Wireless D2D caching networks can be a desirable alternative for OTT service providers and telecommunication operators, but the problem is user resistance. User participation is imperative to deliver wireless D2D caching network functionality successfully. Thus, to gain a deeper understanding of user resistance toward wireless D2D caching networks and their underlying sources, this study introduces two perceived cost factors contributing to this resistance and one perceived benefit that mitigates such resistance. Based on an online survey, this study found new theoretical links among perceived costs and benefits, resistance, and participation intention. The findings reveal that user resistance is predicted by perceived costs, encompassing resource sacrifices and privacy concerns, whereas perceived benefits—specifically, perceived usefulness—did not significantly influence resistance. This implies that telecommunication operators should prioritize market requirements over technological advantages, emphasizing the potential for successful commercialization of wireless D2D caching networks.

## 1. Introduction

Mobile OTT service consumption with handheld and wireless devices has become a part of anytime, anywhere communication in our everyday lives [1]. In particular, mobile OTT service has enjoyed a boost as time spent staying at home has become longer after the COVID-19 pandemic [2]. However, this trend has far-reaching consequences. In recent years, the growth of mobile network traffic has reached unparalleled levels due to the rapid expansion of OTT video content streaming services.

The Korean government has removed mandatory quarantine regulations, which might change the OTT consumption environment and mobile network traffic situation. According to KCC’s research [3], people consume OTT video content not only at home but also in the workplace, school, and transportation, especially during weekdays. The implication is that people use mobile OTT services for both social and outdoor activities. Thus, as the time spent outside the home increases as life returns to normal, OTT video content consumption in the non-residential area will increase.

In Korea, OTT platforms rely on telecommunication operators’ broadband connections for streaming video content [4]. To entice OTT subscribers, telecom operators introduced mobile data plans that include access to mobile OTT services. The COVID-19 pandemic intensified this trend, making mobile devices the primary choice for OTT content consumption [3]. Consequently, the continuous surge in mobile network traffic has become a significant concern for telecommunication operators [5]. According to Ericsson’s report [6], mobile network traffic witnessed a notable 38% increase from the third quarter of 2021 to the third quarter of 2022, primarily fueled by the escalating demand for online video content during the pandemic.

As mobile networks face escalating strain from the surge in OTT content streaming, fiber-to-the-premise (FTTH), known for its high broadband capacity and speed, is recognized as a viable option to alleviate mobile network overloads. Previous research demonstrated that limitations in high-speed mobile networks and issues with unstable video quality are key drivers of individuals’ willingness to adopt FTTH [7]. The study also emphasized that the availability of movie content on OTT platforms significantly influences people’s decision to adopt FTTH connections. This is attributed to the characteristics of movie content, which often entails large file sizes and favors high-definition quality. Consequently, watching such content necessitates a significant amount of data, making it more feasible to afford with an FTTH subscription. Notably, Korea stands out with one of the world’s highest levels of fiber-to-the-premise (FTTH) penetration [8]. Korea is implementing FTTH based on the cost-effective and energy-efficient Passive Optical Network (PON) technology. This high-speed internet deployment ensures robust signals, minimizing the impact on Wi-Fi speed and quality when multiple devices connect simultaneously [9]. Nevertheless, in recent years, mobile network traffic has grown at a rate that existing mobile communication technology cannot keep up with, making it difficult to improve the quality of service. The proliferation of OTT services, competing to expand their subscriber base, has resulted in a substantial surge in mobile network traffic. Historically, mobile communication relied on the infrastructure of cellular networks, requiring telecommunication operators to deploy additional base stations, establish more access points, or expand network bandwidth to enhance performance [5,10]. Nevertheless, limitations in antenna performance, the financial burden of investment, and constrained resources have made it increasingly challenging to improve the functionality of wireless mobile networks. Even though Korea launched the first instance of commercial 5G service in the world in April 2019, consumer complaints are rising over the communication quality of 5G service [11]. Telecommunication operators suffering from mobile data traffic seek technological alternatives to deal with the increasing demand for data.

Accordingly, wireless D2D caching networks emerged as one of the reliable alternatives for addressing mobile network traffic problems [5]. Wireless D2D caching networks indicate a technology that pre-stores frequently requested content in the mobile device storage of a user expected to arrive at the peak, anticipating user demand, and proactively caches content for efficient delivery [5]. The benefits include a reduction in network traffic overload and related overheads [5,12], providing users with high-quality OTT video content, reduced latency, and stable transmission. Therefore, it is rational to think that wireless D2D caching networks will play an essential role in dealing with excessive mobile network traffic. Despite the potential benefits, the successful commercialization of wireless D2D caching networks hinges on user participation, involving the sharing of personal information and mobile device resources. Therefore, mobile OTT service users may resist adopting wireless D2D caching networks on mobile devices and consume their private resources. An inherent tension exists between an individual’s rationality and the whole network’s welfare, reducing the viability of wireless D2D caching networks.

Since it is difficult with the current mobile network technology to properly support the mobile OTT service when moving or staying outside, wireless D2D caching networks offer a good option for OTT service providers and telecommunication operators. However, a significant hurdle to their widespread adoption is user resistance. To identify influential factors contributing to user resistance, this study employs a structural equation model (SEM). The model incorporates two perceived costs—privacy concerns and resource sacrifice—associated with resistance to wireless D2D caching networks, and one perceived benefit—perceived usefulness—which mitigates such resistance. Encompassing user resistance and intention to participate in these networks, the model takes into account the characteristics of wireless D2D caching networks and user rationality. The result from this SEM analysis can provide a deep understanding of users’ concerns about adopting wireless D2D caching networks and significant insights into what aspects of this technology need emphasis or supplementation to succeed in the mobile OTT service market.

This paper is organized as follows. Section 2 presents the research background, exploring mobile network-related issues and technical advancements. Based on previous work addressing mobile network and OTT-related issues in the user context, Section 3 proposes a research model with four research hypotheses. Section 4 explains the research methodology. Section 5 reports the results of the analysis. Section 6 presents our conclusions along with some implications and limitations.

## 2. Research Background

### 2.1. Technical Aspects about Issues and Advancements in Mobile Networks

#### 2.1.1. Mobile OTT Services and Network Traffic in Korea

OTT services, which enable users to stream online video content, have gained significant popularity in Korea [13]. These services offer the convenience of watching video content at any time and on any device, leading many users to shift away from traditional cable TV subscriptions [14]. In Korea, there is intense competition among global players like Netflix, Apple TV, and Disney+, and local providers such as Coupangplay and Tving, all delivering content through telecom operators’ broadband connections [4]. Telecommunication operators have even bundled OTT subscriptions with mobile data plans to attract subscribers. As a result, most Korean OTT users rely on mobile data networks to access content via their smartphones [2]. They do so in various locations, including schools, workplaces, restaurants, and public transportation [3].

Mobile OTT video streaming significantly contributes to mobile network traffic congestion [5]. In Korea, between October and December 2021, Google and Netflix’s mobile video streaming services alone accounted for 27.1% and 7.2% of total mobile network traffic, posing a substantial challenge for telecommunication operators [15]. To address this issue, SK Broadband, a prominent Korean internet service provider, took legal action against Netflix, seeking compensation for increased network traffic and maintenance costs due to a surge in Netflix subscribers [16]. In June 2021, a Seoul court ruled in favor of SK Broadband, holding Netflix responsible for network congestion and ordering compensation [17]. Netflix, however, contested the ruling on net neutrality grounds, prolonging the legal battle. In September 2023, SK Broadband and Netflix resolved their differences, dropping all lawsuits and opting for a partnership. Still, seven bills are before the Korean National Assembly regarding ISPs imposing network usage fees on CPs.

Over time, users have consistently expressed their desire for faster and more reliable transmission, higher image quality, and a more convenient OTT service experience, as evidenced by previous studies [18,19,20,21]. Meeting these demands requires mobile networks to maintain stable and speedy connections. However, existing mobile communication technology faces challenges in coping with the surging demand for mobile network traffic [5,10].

The current 5G adoption rate among mobile subscribers in Korea is approximately 38%, with anticipated growth [22]. Nevertheless, the coverage remains limited, and network quality remains subpar [11]. Consumers cannot fully enjoy the service despite paying premium rates for 5G mobile data plans [11]. Additionally, the cost and resource constraints pose challenges in expanding the network bandwidth or deploying more base stations [5]. So, companies are seeking technological alternatives to deal with the exploding OTT consumption and mobile network demand.

#### 2.1.2. The Advancement of Wireless and Mobile Communication Technology

The communications industry has witnessed rapid advancements in wireless and mobile communication technologies. These developments have captivated the media and public, making smartphones indispensable tools in our daily lives. With the current generation of wireless LANs, based on IEEE 802.11 standards, wireless and mobile communication technology has emerged as a substitute for and complement to wired networks across various domains, encompassing homes, schools, and offices [23].

**Figure 1:**
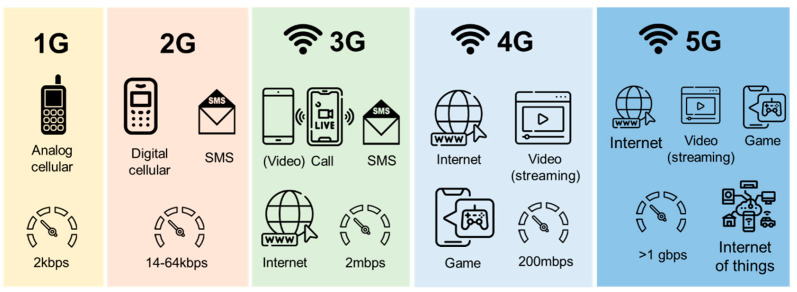
Generational evolution of mobile communication network.

Mobile communication technology, enabling information exchange between individuals and devices, stands at the forefront of communications. It has evolved from 0G to 5G. Introducing 1G (1980–1990) and 2G (1990–2004) allowed mobile communication worldwide. Then, 3G, adopted widely from 2004 to 2010, provided greater bandwidth and global standards [24]. Since 2009, 4G has improved performance through spectrum use and multiplexing. Notably, 3G and 4G enabled mobile internet access, voice calls, and HD video calls. Since 2019, global adoption of 5G services has surged, offering superior data rates, lower latency, and broader device connectivity [25]. This advancement has unlocked possibilities like the Internet of Things and HD multimedia streaming, poised for widespread adoption across diverse fields. Figure 1 illustrates the generational evolution of mobile communication network.

In the evolution of wireless and mobile communication technology, alternative technologies have emerged to address cellular network limitations. Ad hoc networks, a well-established alternative, consist of communication devices (nodes) that establish connections independently, without fixed infrastructures or predefined links [26]. These networks adapt to changing connectivity and link conditions due to node mobility and power control practices, making them valuable when traditional infrastructure is absent or unsuitable, especially in urgent scenarios [26]. Nevertheless, ad hoc networks face challenges, notably scalability: the ability to serve a large population while managing mobility effectively [26,27]. Addressing this issue requires supplementary routing technologies [28]. Moreover, energy efficiency, security concerns, hostile environments involving losses and noise, and sporadic connectivity pose ongoing challenges for ad hoc networks [26,29].

**Figure 2:**
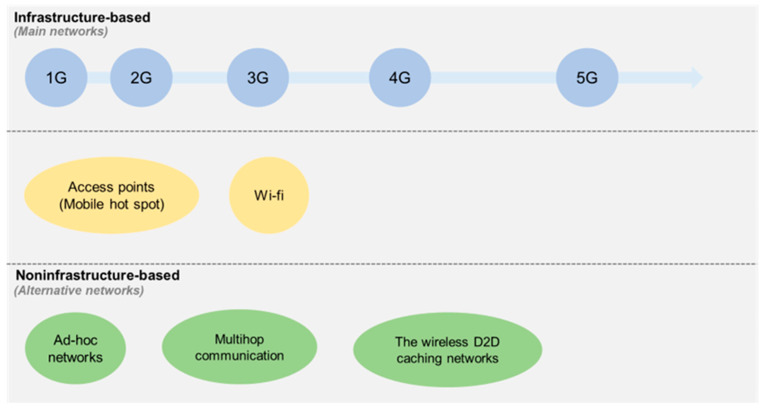
The position map of wireless and mobile communication technologies.

As an alternative technology, multihop communication establishes wireless networks in areas lacking traditional infrastructure [30], such as disaster-stricken regions. It offers enhanced spectral efficiency, increased transmission rates, reduced power usage, and lower communication costs [31]. However, multihop communication faces practical challenges. Intensive data exchange in specific network areas can hinder optimization [30]. Security concerns arise from unauthorized mobile users and potential traffic disruptions [32]. Privacy issues also affect multihop communication, including location and identity exposure [30]. Figure 2 illustrates the position map of wireless and mobile communication technologies.

Alternative telecommunications technologies like ad hoc networks and multihop communications offer technological advantages but face limitations that hinder full commercialization. Meanwhile, advancements in mobile communication continue to address challenges. In this context, wireless D2D caching networks emerge as promising alternatives. Their successful commercialization hinges on understanding their strengths, weaknesses, and factors crucial to users. This study aims to identify key criteria for mobile OTT service users in adopting wireless D2D caching networks, forming a foundation for a successful commercialization strategy.

#### 2.1.3. Wireless D2D Caching Networks

Wireless D2D caching networks refer to a technology that pre-stores popular content in the mobile device storage and transmits it over D2D links among users arriving at the place at peak times rather than using cellular access links [5]. Within cellular networks, user devices form data connections through government-assigned licensed spectrums exclusive to telecommunication operators. Traffic is generated on base station-to-user links [33]. In contrast, D2D communication operates under the control of individual mobile devices. At one point, peer-to-peer (P2P) networks gained widespread popularity on the internet as a means of alleviating the burden on network traffic [34]. In P2P networks, a collective network of nodes is formed among peers, with participants contributing a portion of their resources, such as disk storage or network bandwidth, directly to one another. This eliminates the requirement for centralized coordination by home servers [34,35]. To optimize the streaming process, popular video content is often stored in proxies or intermediate servers, facilitating direct streaming to users instead of relying solely on home servers [34]. D2D communication shares similarities with P2P networks but involves multiple direct data transmissions between pairs of nearby devices. In wireless D2D caching networks, a D2D transmitter retrieves requested data from mobile device memory and concurrently retransmits it directly to a D2D receiver [33]. Short-range communication technologies, such as Wi-Fi Direct and Bluetooth can be leveraged for D2D communication [33]. 

When it comes to accessing content, in Data Center Networks (DCNs), the primary method involves centralized data centers, where users submit requests for content retrieval. This centralized approach often leads to bottlenecks and delays, particularly during peak traffic periods [36]. Additionally, packet contention can occur at network routers, where packets from multiple users or applications vie for transmission through shared network resources simultaneously [37]. Similarly, D2D communication can face packet contention issues, especially in environments with high device density. Nevertheless, the implementation of a cooperative caching strategy that leverages user dynamics across multiple cells can effectively address this challenge [38].

**Figure 3:**
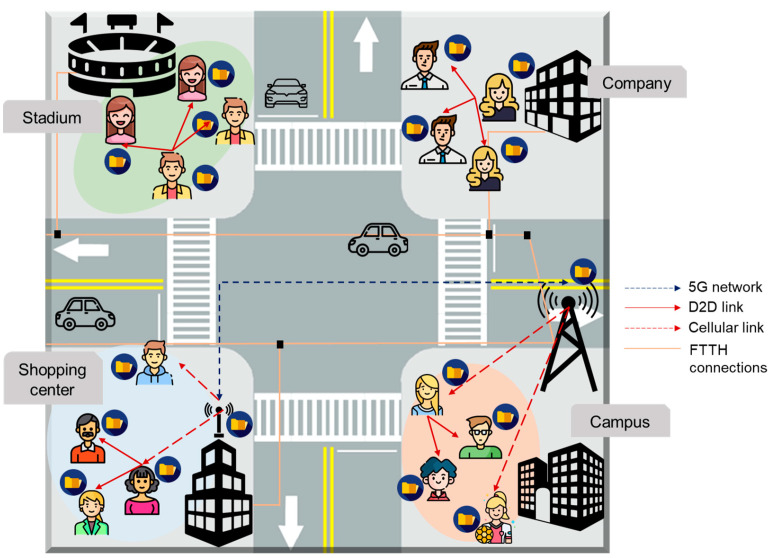
Illustration of various applications of the wireless D2D caching networks.

Since wireless D2D caching networks enable users to engage in direct D2D communication regardless of whether the base station is nearby as shown in Figure 3, it can minimize the overloads of a mobile network. Once a user agrees to adopt these networks, the content caching and transmission processes are automatically executed based on the mechanisms designed by the service provider, for example, a telecommunication operator. Even when the transmitter device communicating with a receiver device becomes disconnected due to distance, the continuity of watching OTT video content remains largely uninterrupted, as the receiver device can establish a direct connection with another nearby transmitter device. 

Technically, wireless D2D caching networks have multiple advantages. Most of all, this technology significantly decreases mobile traffic loads during rush hours, particularly in the case of the high-density user population, by interconnecting mobile device storage and users with wireless caching [10]. It enables fast and stable content delivery by lifting the load on the mobile network traffic using cache memory instead of network resources such as bandwidth [10]. Accordingly, this technology efficiently reduces network-related overloads and saves related costs [12]. Furthermore, there has been discussion surrounding integrating artificial intelligence (AI) into wireless caching to enhance content caching efficiency [39]. Network providers can accomplish this enhancement by accurately predicting user data requests and understanding data popularity profiles. AI-based wireless caching is a promising solution to address challenges in forthcoming mobile networks, including issues like redundant data transmission and data access delays [39]. For instance, the authors of [40] explored AI-empowered wireless caching in the context of vehicular networks. Their study delineated three primary tasks within this framework: predicting content popularity, determining optimal content placement in caches, and efficiently retrieving content from them.

As innovative alternative telecommunication technology for data transmission, wireless D2D caching networks provide various benefits for mobile OTT service users. So far, most OTT service platforms have tried to create and deliver high-definition video content [20]. However, when users are outside or moving, it is difficult to maintain high picture quality because of unstable connections. The transmission technique employed in wireless D2D caching networks can enhance picture and video quality by ensuring stable transmission [41]. Numerous D2D caching studies introduce various methods for encoding video content at quality levels that align closely with user expectations [41]. 

Furthermore, because wireless D2D caching networks deliver the content directly to other devices without separate communication with the server [41], it is considered a reliable technological alternative that significantly reduces content transmission latency, increases the probability of correct content delivery, and thus assists with smooth online video streaming [42]. 

Despite these merits, users bear some costs when adopting wireless D2D caching networks on their mobile devices. Even though vendors have not yet commercialized wireless D2D caching networks, it is a highly plausible future option for telecommunication operators suffering from considerable network headaches. Therefore, it is meaningful to consider costs users feel unwelcome. First, the privacy issue represents the potential cost of adopting wireless D2D caching networks. Second, consuming users’ mobile devices’ resources is another possible cost. These issues could be impediments for users.

## 3. Research Hypotheses and Model

### 3.1. User-Related Aspects about Issues and Advancements in Mobile Networks

#### 3.1.1. Technology Acceptance and Resistance

As technology has continuously developed and integrated into our lives, users’ decisions to accept or reject new technology become a critical question [43]. Accordingly, user acceptance of new technology is one of the essential constructs in numerous studies of information systems (IS) and new media. Studies in these areas have produced multiple theoretical models based on technology, psychology, and sociology, and they usually show high explanatory power for individual intention to participate in technology [44,45]. 

Historically, there have been consistent theoretical efforts to explain user behavior. Fishbein and Ajzen [46] initially presented the theory of reasoned action (TRA), which demonstrates that the intention to perform a particular behavior precedes the actual behavior, and attitudes to behaviors and subjective norms determine this intention. The technology acceptance model (TAM), which evolved from TRA, is another theory to elucidate how users accept and use technology. TAM indicated that perceived usefulness and ease of use influence technology usage [44]. However, the diffusion of innovation theory (DIT) argues that a user’s decision to accept and use technology is a function of perceived attributes of innovations, social norms, and individual characteristics [47]. Scholars have applied these theories diversely to examine traditional technologies (e.g., personal computers, automatic teller machines, and e-mail) and new or innovative technologies such as e-mail [48], cellular telephones [49], mobile commerce [50], wireless internet [51,52] and e-learning [53]. 

As various fields widely introduce new technologies, researchers and practitioners have tried to identify which factor influences users’ beliefs and attitudes on the technology acceptance decision [43]. Researchers have employed considerable external variables in their studies to understand and improve the predictability of behavioral intention for technology acceptance [43]. Despite these efforts, the resistance restraining the user’s decision to accept and use technology is a low-profile construct from theories [30]. 

User resistance to new technology is the intentional acts or commissions that oppose changes related to new technology implementation [54,55]. There may be a situation where a user wants to use technology but hesitates because of worries about the quality of technology or resource restrictions. Since change frequently accompanies new technology adoption, user reactions to this change might differ [56]. However, the technology acceptance literature has not paid much attention to user resistance to new technology. Even though researchers acknowledged that user resistance to accepting and using new technology is an important dimension, they treated it as a black box [30]. Thus, most researchers have focused heavily on positive and successful reactions to new technology. They assumed that innovations are primarily beneficial and helpful to improve the system of society [57,58]. These biased ideas quickly led to the conclusion that most society members embrace innovations.

According to Kuisma et al. [59], scholars should consider user resistance to new technology a natural factor in a decision process rather than a necessary factor leading to hesitation to adoption. User resistance occurs when a user prefers existing practices or perceives new technology as costly. Markus [60] elucidated that users’ internal factors and poor system design and interaction with specific system design features related to the context of technology use contribute to resistance to new technology. Given this, some researchers tried to find constructs that make users reluctant to use technology and empirically tested them. Studies including human behavior, communication, psychology, organization science, and strategic management have found external variables associated with user resistance. 

In the field of IT, researchers have explored uncertainty [61,62], lack of involvement in the change [61], lack of necessary capabilities [63], and switching costs [63,64]. Previous studies also examined the effects of disconfirmation of products. The studies found that disconfirmation occurred when perceptions of product performance after the trial differed from pretrial beliefs about the product, affecting people’s behaviors [65]. Computer anxiety is another belief construct deployed by researchers. Researchers have pointed to many human factors that elucidate why individuals with computer anxiety and a negative attitude toward computers tend to shy away from and resist learning about computers [66]. For instance, Pavlou et al. [67] focused on the uncertainty of the online environment that made consumers reluctant to engage in online exchange relationships and investigated the sources of uncertainty in technology acceptance. 

However, such studies did not explain why users hesitate since they dealt with those constructs as black boxes [30]. Additionally, they also have limitations when applied in a very dynamic context. Recently, Kang and Kim [30] examined different perceived costs that generate resistance to multihop communications. They found theoretical links between perceived resistance and participation intention. Wireless D2D caching networks are an unfamiliar communication method in that users will be not only transmitters but also receivers. Given this, user resistance could apply in wireless D2D caching networks. The participation of unfamiliar users, rather than established telecommunication operators, could lead to user resistance against wireless D2D caching networks. Therefore, a model that explains users’ participation in wireless D2D caching networks should include resistance as an attitude.

As explained earlier, numerous theories and empirical tests of technology acceptance have suggested that intention is a strong and significant predictor of actual behavior. As wireless D2D caching networks are not yet widely commercialized, this study employs “intention” to estimate actual participation. Previous studies used various terms concerning technology acceptance intention, such as intention to use or accept. However, this study adopted the term “participation intention” in the sense of participating in wireless D2D communication. Most models have indicated a positive relationship between attitudes and intentions. However, in the context of user resistance, we expected that there is a negative relationship between resistance (attitude) and participation intention. For example, a previous study by Pavlou et al. [67] discovered that uncertainty, a negative attitude, negatively influences participation intention in online transactions. Kang and Kim [30] identified that perceived resistance and participation intention have a negative relationship. Based on the above discussion, this study tried to include proper constructs and their sources to better explain participation in wireless D2D caching networks. It hypothesizes the relationship between resistance and participation intention as follows.

**Hypothesis 1.**  *Resistance will negatively affect participation intention in adopting wireless D2D caching networks on mobile OTT service user devices*.

#### 3.1.2. Perceived Costs

In technology acceptance research, perceived cost is the cost incurred in technology adoption [68]. Some studies operationalize perceived cost as perceived risk [69,70]. The perceived cost works as a barrier to new technology adoption [68,71]. In other words, if users perceive the cost of adopting a new technology as relatively critical and high, they do not think adopting a new technology is justifiable and resist participation [68,72].

Valence theory explains the relationship between perceived cost and intention to use or adopt new technology [73,74]. This theory insists that a product or service’s evaluation is a function of desirable benefits with positive valence and undesirable costs (risks) with negative valence. If the perceived costs or risks outweigh the perceived benefits, resulting in a net negative valence, the user may not follow through with their intention to purchase or use. Information technology studies widely use this net valence approach. For instance, Featherman [75] investigated the individual adoption intention of internet-based e-payment systems using perceived benefits and risks based on this framework. Zainab et al. [72] also examined the role of perceived cost and perceived benefit-related factors in e-training adoption in the Nigerian civil service.

Perceived cost or risk, which makes a person feel uncertainty about adopting a technology due to the expected negative and unpleasant consequences, such as threatening core values or existing habits, can be a significant cause of resistance [69,76,77]. However, despite frequent use, technology acceptance research has not considered the cost or risk concept as a major construct [30]. In this study, we assumed that users’ perceived costs could induce resistance to wireless D2D caching networks, as they develop a negative attitude when perceiving costs or risks related to new technology. Accordingly, this study assumed that perceived cost is a critical source of resistance to adopting wireless D2D caching networks on mobile OTT user devices. Furthermore, considering the various issues in wireless D2D caching networks, this study specified perceived cost by proposing two types of perceived cost: (1) privacy concerns and (2) sacrifice of resources.

##### Privacy Concerns

Privacy concerns revolve around the potential intrusion into users’ privacy due to the open nature of wireless D2D caching networks [78,79]. D2D communication might share certain information, such as users’ location, content viewing history, and content preferences, to enhance the hit ratio when communicating with other users’ mobile devices [78]. Especially, AI-based wireless caching poses a challenge regarding user authentication, as it contains numerous users with private and confidential information in their profiles [39]. When utilizing learning tools for training, there is a risk of training data containing sensitive personal information related to social connections and proximity relationships [39]. The leakage of such data can lead to security and privacy threats for users, and this privacy constraint can hinder their acceptance of wireless D2D caching networks [79]. Indeed, VanSlyke et al. [80] discovered that many individuals wish to minimize the risk of personal information abuse by online companies. In addition, Nam et al. [81] mentioned that users’ heightened concerns regarding the misuse of personal information online can create hesitation in granting permission to use their personal data. Thus, privacy concerns caused by wireless D2D caching networks are a significant cost that mobile OTT service users face when considering adopting this technology. Therefore, this study’s second hypothesis is:

**Hypothesis 2.**  *Privacy concerns will positively affect resistance to adopting wireless D2D caching networks on mobile OTT service user devices*.

##### Sacrifice of Resources

The sacrifice of resources is another potential cost when adopting wireless D2D caching networks. During the implementation of wireless D2D caching networks, the size of content and the allocation of mobile device storage play crucial roles [82]. For instance, users must allocate some of their mobile device storage to cache content. However, as mobile technology and applications continue to advance, a substantial amount of storage on users’ mobile devices is already occupied [83]. Previous studies have indicated that this trend will likely intensify due to the progressive evolution of mobile apps’ functionality and technologies like AI, augmented reality (AR), and 4K graphics. Consequently, the additional storage demands imposed by wireless D2D caching networks could be expensive for users who have already stored substantial amounts of data, including photos, videos, and apps, on mobile devices with limited storage capacity.

Furthermore, the operation of wireless D2D caching networks, which involves pre-storing frequently requested content in device storage and delivering it upon request, can result in increased battery drainage during D2D communication. This aspect becomes crucial as maintaining a high battery level is a sensitive concern for most smartphone users [84,85]. Compounding the severity of this issue is that smartphones have become essential tools in people’s daily lives, serving purposes such as social networking and shopping. Consequently, sacrificing the battery represents a substantial cost for users considering the adoption of wireless D2D caching networks on mobile devices. Based on this discussion, this study assumes that users of wireless D2D caching networks tend to have high resistance if they expect more sacrifice of more resources such as device storage or battery.

**Hypothesis 3.**  *Sacrifice of resources will positively affect resistance to adopting wireless D2D caching networks on mobile OTT service user devices.*

#### 3.1.3. Perceived Benefit

The current study dealt with variables related to users’ benefits in return for sacrificing resources when adopting wireless D2D caching networks. User resistance may be inversely proportional to incentives. Thus, this study included the factor related to incentives corresponding to the cost: perceived usefulness. It is a surrogate for incentives obtained by adopting wireless D2D caching networks regarding service use and enjoyment.

##### Expected Usefulness

Perceived usefulness refers to the degree to which a person believes using a particular technology would improve their performance [44]. Many studies have deployed new technology’s perceived usefulness as an important factor when describing user resistance [55,86,87,88]. These studies have confirmed that users’ perceived usefulness is significantly related to user resistance to adopting new technology. Wireless D2D caching networks allow users to experience improved picture quality, transmission stability, and speed through wireless D2D communication without going through the base stations of mainstream telecommunication operators. Furthermore, by integrating AI into wireless caching, optimal data caching, retrieval, and transmission can be achieved by improving the accuracy of predicting the content users will request [39]. Since these factors will likely significantly impact users’ welfare when consuming mobile OTT services outdoors or while moving, it can provoke users’ perceived usefulness. Considering that perceived usefulness encompasses users’ expectations for improved effectiveness, productivity, and performance, the current study defined perceived usefulness as the belief that participation in wireless D2D caching networks will enhance a user’s effectiveness, productivity, performance, and welfare. Hence, this study proposes the following hypothesis.

**Hypothesis 4.**  *Perceived usefulness will negatively affect resistance to adopting wireless D2D caching networks on mobile OTT service user devices*.

**Figure 4:**
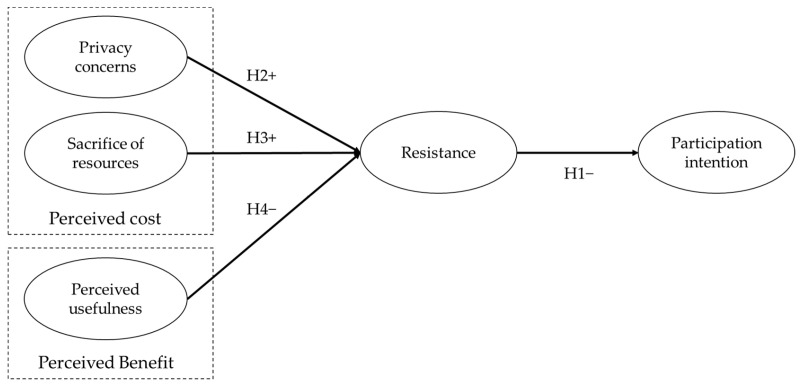
SEM model for participating in wireless D2D caching networks.

In summary, even though the success of wireless D2D caching networks depends greatly on user participation, this cannot be guaranteed because of inherent user resistance stemming from perceived costs. Thus, this study investigates what factors make users resist wireless D2D caching networks. Simultaneously, this study also deals with perceived benefits to examine whether the incentive offsets user resistance. The proposed framework in this study integrates the literature and hypotheses described above (Figure 4).

## 4. Research Methodology

### 4.1. Data Collection and Analysis

Data were collected for Korean samples through online surveys. Korea is one of the leading countries in mobile communication technology, and most OTT service users consume video content through their smartphones. Thus, we recruited participants who subscribed to mobile OTT services through an online survey company from 7 to 9 June 2022. In the online questionnaire, participants provided their demographics, attitudes, and intentions to adopt wireless D2D caching networks on mobile devices. Because wireless D2D caching networks are not yet commercialized, this study first explained the concept and characteristics of the technology in the form of cartoons before the survey. The survey instrument was constructed based on established measures of constructs from marketing and IS literature, adapted to the context of our proposed model. All items were anchored on a seven-point Likert scale ranging from “1 = strongly disagree” to “7 = strongly agree”. Table A1 in the Appendix shows all items used in our survey. This study adopted SEM using Smart PLS (partial least squares) 4.0 to analyze the theoretical propositions because of its efficiency for simultaneously testing multi-staged causal relationships [89].

### 4.2. Sample Characteristics

This study analyzed 312 responses. The profiles of the respondents are in Table 1. In summary, respondents subscribed to an average of 2.81 mobile OTT services and subscribed to Netflix the most. Respondents spent an average of KRW 16,945.71 (USD 12.73) (the Korean Won or KRW is the monetary unit of Korea. On 19 May 2023, USD 1 was approximately KRW 1330.89) monthly on subscription fees.

### 4.3. Testing of Measurement Model

Cronbach’s alpha and the composite reliability (CR) test examine the internal consistency within a construct. As shown in Table 2, all constructs had a value above the threshold of 0.7 for Cronbach’s alpha and CR, adopted by Werts et al. [90]. Convergent validity reflects the extent to which the indicators of a construct strongly correlate to each other than to indicators of other constructs. To test convergent validity, CR and average variance extracted (AVE) are examined. It is acceptable for an individual item factor loading to be greater than 0.5, CR to exceed 0.7, and AVE to exceed 0.5 [91]. As shown in Table 2 and the Appendix in Table A2 and Table A3, all values are above the marginal standard. Thus, the convergent validity of the construct is adequate. This study examined the table correlation of constructs and the latent square root of AVE to test discriminant validity. It shows that measurement items load highly on their theoretically assigned constructs and do not load on other factors. To satisfy discriminant validity, the square root of AVE should be greater than the correlations between different constructs [92]. As Table A3 in the Appendix presents, the square root of AVE for each construct in this study exceeded the correlations between the construct and other constructs. Thus, the factors have discriminant validity.

## 5. Analysis and Results

This study hypothesized and tested key variables reflecting the context of wireless D2D caching networks. Previous studies have limitations in that they cannot fully explain the dynamic context of adopting new technology, such as wireless D2D caching networks, since they have not paid much attention to resistance and perceived cost that would affect an individual’s intention to adopt new technology. Thus, they potentially mislead in explaining users’ participation intention. Indeed, as many previous studies focused on users’ positive reactions to adopting new technology, they formed the unwarranted belief that people will voluntarily welcome new technology [93]. To fill the research gap, the current study encompassed negative (cost) and positive (benefit) aspects of wireless D2D caching networks and tried to discover which factor significantly influences user resistance.

**Figure 5:**
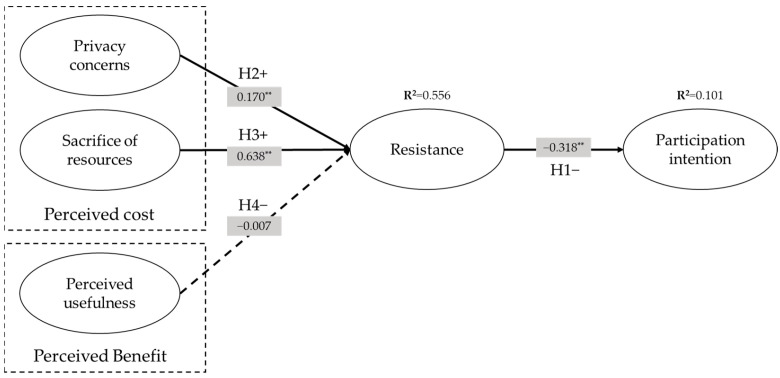
SEM results for research model. ** *p* < 0.01.

To verify the hypotheses, this study employed SEM analysis. Our research model could explain 10.1% of the variance in participation intention and 55.6% in resistance. Table 3 and Figure 5 show our research model with a summary of the results following the testing of hypotheses; a boot-strapping procedure was used to confirm the significance of the path coefficients. The analysis resulted in support for three of the four hypotheses. First, resistance significantly negatively affected participation intention (supporting H1), validating this paper’s key position that a resistant attitude impedes participation in wireless D2D caching networks. Second, regarding the two types of perceived cost as the source of resistance, both privacy concerns and sacrifice of resources were significantly related to resistance (supporting H2 and H3). However, regarding perceived benefit, the effect of perceived usefulness did not have a statistically significant relationship with resistance (rejecting H4).

## 6. Discussion and Conclusions

### 6.1. Key Findings and Implications

As discussed earlier, the escalating growth of mobile OTT services and the resulting burden on mobile data traffic are interconnected challenges. To deliver a consistent and satisfactory mobile OTT service experience, reliable alternatives must be explored. Consequently, wireless D2D caching networks have emerged as a dependable solution, given their economic advantages in reducing mobile network-related expenditures. With a high likelihood of telecommunication operators seeking to commercialize this technology, the findings of our study underscored the importance of prioritizing an understanding of perceived costs and resistance toward wireless D2D caching networks. In other words, successfully deploying wireless D2D caching networks necessitates a focus on market requirements rather than solely relying on technological advantages, emphasizing potential possibilities. Companies should develop innovative policies and strategies in related sectors to align with technological evolution. Additionally, addressing privacy concerns is crucial in the utilization of wireless D2D caching networks, as it may evolve into a more significant social issue. Therefore, network providers and policymakers must proactively manage privacy considerations during the commercialization of wireless D2D caching networks.

The empirical results of this study shed light on user resistance that can lead a user to a negative psychological attitude. It found attitude a key variable in predicting mobile OTT service users’ participation intention in wireless D2D caching networks. It also found that perceived cost is vital in explaining users’ resistant attitudes toward wireless D2D caching networks. Thus, the results of this study may enhance understanding of a fundamental set of constructs, such as resistance and perceived cost, that technology acceptance literature has overlooked. If the proven theoretical links among perceived benefits, positive attitudes, and behavioral intention in technology acceptance theories are complemented by additional theoretical links among perceived costs, resistance, and behavioral intention, it can enhance the explanatory power and understanding of user’s perceptions and intentions toward new technology, especially when benefits and costs of technology are mixed and uncertain [30].

Moreover, the findings of this study lend support to the “what” and “how” regarding user hesitation in participating in wireless D2D caching networks. Above all, this study found that resistance to wireless D2D caching networks decreases participation intention. This finding supports the argument that resistance is crucial in understanding human behavior toward change [30]. Among perceived costs, the sacrifice of resources is a stronger predictor of user resistance. This result is not surprising because previous literature noted that users consider maintaining sufficient smartphone storage and battery level critical [83,84,85]. Thus, if users think they do not have enough storage or charge in the battery, they may stay with existing mobile communication technologies. In this sense, telecommunication operators must improve wireless D2D caching networks’ reliability and energy efficiency to consume less battery.

Privacy concerns also significantly affected resistance. This result is consistent with previous studies describing that privacy concerns negatively influence users’ intentions to use. However, compared to the sacrifice of resources, privacy concerns showed a relatively weaker influence on resistance. A plausible reason for this finding might relate to Korean users’ relative indifference to privacy issues. For instance, Park et al. [94] found that the impact of privacy costs on adopting e-commerce is stronger for American users than for Korean users. This result is interpreted in that individuals from a collectivist culture, such as Koreans, place relatively less importance on privacy concerns than individuals from an individualist culture, such as Americans [95]. Second, the OTT platform already uses information such as OTT video content viewing history and preferences required to utilize wireless D2D caching networks to provide customized recommendation services for users. Thus, users’ resistance toward providing such information may be less than concerns about sacrificing smartphone battery and storage related to direct and essential use in daily activities. However, there is no doubt that privacy concerns are crucial in provoking user resistance.

Regarding perceived benefit, perceived usefulness did not have a statistically significant effect on resistance. This finding implies that factors related to incentives obtained by adopting wireless D2D caching networks do not strongly influence offsetting user resistance. A plausible reason for the different result than expected is that this study’s measurement of items of perceived usefulness are somewhat abstract and macroscopic. This approach might not seem effective in overcoming resistance compared to the direct and noticeable costs, failing to generate a net positive valence.

In the mobile OTT and telecommunication market, it is expected that wireless D2D caching networks, which are non-infrastructure-based networks among users’ mobile devices, will play an important role. Although wireless D2D caching networks face several obstacles and cannot meet all the demands of service providers and users as discussed in this study, deploying this type of wireless communication technology will provide many opportunities. To capitalize on these opportunities, encouraging user participation by reducing perceived costs and resistance is essential. Thus, the results of the current study have several implications.

Most of all, telecommunication operators should try to minimize user resistance to wireless D2D caching networks by reducing users’ perceived costs. First, companies should reward participants of wireless D2D caching networks for their resource sacrifice. As smartphone users consider their smartphones a gateway to their daily lives and that mobile apps’ functionality and technology continuously evolve, resource issues will be more sensitive to users. If users resist participating in wireless D2D caching networks due to constraints related to device or data resources, telecommunication operators may need to invest in establishing more infrastructure. The anticipated benefits of wireless D2D caching networks may include telecommunication operators’ reduction of capital expenditures (CAPEX) and operational expenditures (OPEX). They would circumvent unnecessary investments in mobile network infrastructures and associated maintenance costs, as well as reduce energy consumption. Additionally, wireless D2D caching networks may enable telecommunication operators to retain their subscriber base since the networks do not impose limitations on service offerings [96,97]. Recognizing these advantages, telecommunication operators should set up and implement incentive systems that include direct or financial (e.g., data plan cost cut) and indirect or psychological (e.g., title or reputation) factors to induce higher user interest and participation. 

Second, telecommunication operators can create cohesion among users because users would be less resistant to wireless D2D caching networks in a cohesive atmosphere [30]. For instance, telecommunication operators may organize a virtual community where users can share relevant information and form social intimacy. Alternatively, they can collaborate with large organizations like campuses or workplaces to leverage offline cohesion within the same organization. It may be possible to partner with big fan clubs and sports societies. Since they share similar interests and gather regularly, increasing the hit ratio of networking and energy efficiency would be easy. Achieving critical mass would help improve users’ hospitality and settlement of wireless D2D caching networks.

Third, since users are more inclined to adopt technology-based services when they are easily accessible and reliable [98], telecommunication operators should regard accessibilities and reliability as preconditions for the utilization of wireless D2D caching networks. To fulfill these preconditions, operators could, for instance, introduce a user-friendly app facilitating the (de)activation of wireless D2D caching networks. They may collaborate with mobile device manufacturers to integrate this feature into a quick setting window (similar to Wi-Fi) to make it more accessible. The development of a more flexible short-range communication technology beyond Wi-Fi Direct and Bluetooth would further enable the reliable delivery of content through D2D communication [33]. Additionally, strategic collaboration with OTT platforms to analyze content data and predict frequently requested content in specific areas could significantly enhance user willingness to participate in wireless D2D caching networks. Accordingly, although telecommunication operators play an essential role in commercializing this technology, the cooperation with related parties like mobile device manufacturers and OTT platforms would be crucial, too.

Meanwhile, as wireless D2D caching networks enable users to form their communications networks utilizing personal resources, dependency on access networks provided by telecommunication operators may decline. When there is enough user participation, dissatisfaction with the telecommunication operators’ policies regarding rates and benefits could rise. Telecommunication operators should establish effective management policies and strategies and develop a robust mobile data plan to safeguard their business’s core structure and retain customers.

### 6.2. Limitations and Future Research

This study has several limitations and suggestions for future research. First, there may be a sampling bias since participants for this study were recruited only from Korea. The results will likely vary by country due to differences in social and cultural environments. For instance, since the mobile data plan system or main ways to consume OTT service may differ from those in Korea, users in other countries could have different perceptions and attitudes toward wireless D2D caching networks. Considering and comparing these differences is an interesting topic for future research. 

Second, this study employed only perceived usefulness as a benefit of wireless D2D caching networks. This study’s measuring items of perceived usefulness are somewhat abstract and macroscopic. So, there may be limitations in putting more weight on perceived benefits than perceived costs, which users can feel concretely and immediately. Thus, future studies could present benefits in terms of direct incentives that users can feel, such as payback to receive a certain amount of money or earn points. It would also be interesting to determine which benefit is more promising to persuade mobile OTT service users to adopt wireless D2D caching networks. 

Third, this study used participation intention to estimate actual participation behavior. Even though researchers widely used participation intention as a critical determinant of people’s behavior, intention and actual participation may have different phases in adopting new technology. Thus, future studies could measure participation in wireless D2D caching networks.

Notwithstanding its limitations, this study expands the literature on adopting new technology by examining the effects of factors reflecting the specific context of wireless D2D caching networks and user resistance. Researchers and practitioners must begin recognizing how users’ perceptions of these factors affect their resistance and participation intention toward a new technology. We hope our study will ignite research on adopting new technology and user participation.

## Figures and Tables

**Table 1:** Sample characteristics (N = 312).

Characteristics	Frequency	Valid Percent
Number of mobile OTT service in use		
1	151	48.4
2	92	29.5
3	45	14.4
More than 4	24	7.7
Most used mobile OTT service (multiple response)		
Netflix	213	68.3
Tving	28	9.0
Wavve	26	8.3
Coupangplay	17	5.4
Disney+	15	4.8
Watcha	5	1.6
AppleTV	3	1.0
Seezn	2	0.6
Others	3	1.0
Age		
20 s	78	25.0
30 s	80	25.6
40 s	78	25.0
50 s	76	24.4
Gender		
Male	156	50.0
Female	156	50.0
Occupation:		
Student	28	9.0
Housewife	28	9.0
Office worker	192	61.5
Professional	33	10.6
Self-employed	23	7.4
Other	8	2.6
Education:		
High school	33	10.6
College	241	77.2
Advanced degree	38	12.2
Income (per month, USD):		
≤1000	2	2.1
>1000, ≤2000	4	4.3
>2000, ≤3000	22	23.4
>3000, ≤4000	11	11.7
>4000, ≤5000	13	13.8
>5000	42	44.7

**Table 2:** Descriptive statistics of variables.

Variable Name	Code	No of Items	Mean (Std. Dev)	Cronbach’s Alpha	AVE	Composite Reliability
Privacy concerns	PC	4	5.39 (1.09)	0.903	0.774	0.905
Sacrifice of resources	SR	4	4.86 (1.09)	0.855	0.696	0.859
Expected usefulness	PU	4	4.32 (1.09)	0.929	0.825	0.932
Resistance	RT	4	4.64 (1.06)	0.849	0.689	0.852
Participation intention	PI	4	3.89 (1.33)	0.936	0.839	0.937
Total items		20				

**Table 3:** Direct impact of model: Standardized regression weights.

H	Relations	Std. Estimate	S.E	T-Value	*p*-Value
H1	RT → PI	−0.318	0.069	4.587	0.000
H2	PC → RT	0.170	0.048	3.528	0.000
H3	SR → RT	0.638	0.041	15.432	0.000
H4	PU → RT	−0.007	0.049	0.134	0.893

## Data Availability

The data are available from the corresponding author upon reasonable request.

## Appendix A

**Table A1:** Measurement items.

Construct	Item	Reference
Privacy concerns	It bothers me that my location information, OTT video content viewing history, and preference information may be exposed by adopting the wireless mobile caching networks on my mobile device.	[67]
	I am concerned that telecommunication operators/OTT platforms are collecting too much information (e.g., location information, OTT video content viewing history, and preference information) about me.	
	Agents who pass through my personal mobile device could access other types of information (e.g., SNS, websites, apps, etc.) stored in it.	
	Other external agents could retain my personal information while adopting the wireless mobile caching networks.	
Sacrifice of resources	I do not like to provide my personal mobile device storage to communicate with other OTT service users through wireless mobile caching networks.	[30,99]
	I feel burdened by providing a significant amount of my personal mobile device storage to adopt wireless mobile caching networks.	
	I am worried that I will not be able to use my personal mobile device because of battery shortages when adopting wireless mobile caching networks.	
	I feel burdened by consuming a significant amount of my personal mobile device’s battery to adopt wireless mobile caching networks.	
Perceived usefulness	The wireless mobile caching networks will enhance my satisfaction with using OTT services.	[44]
	The wireless mobile caching networks will help me to easily achieve what I want to do when I use OTT services.	
	The wireless mobile caching networks will reduce time and costs when using OTT services.	
	I will find adopting the wireless mobile caching networks on my mobile device useful in using OTT services.	
Resistance	I feel uneasy when data associated with OTT service are transmitted by wireless mobile caching networks.	[100]
	I insist upon using OTT services delivered through fixed infrastructure (base stations, access points, or bandwidth) rather than wireless mobile caching networks.	
	The wireless mobile caching networks via OTT service users’ mobile devices deserve criticism.	
	I am dissatisfied with the wireless mobile caching networks via OTT service users’ mobile devices.	
Participation intention	I plan to participate as an agent in the wireless mobile caching networks using my mobile device.	[30,44]
	I plan to provide the information necessary to adopt the wireless mobile caching networks (e.g., location information, OTT video content viewing history, and preference information) on my mobile device.	
	I plan to watch OTT video content delivered by others’ mobile devices through wireless mobile caching networks.	
	I will advise others to participate in the wireless mobile caching networks.	

**Table A2:** Cross-loading table for the constructs.

	Privacy Concerns (PC)	Sacrifice of Resources (SR)	Perceived Usefulness (PU)	Resistance (RT)	Participation Intention (PI)
PC1	0.876	0.482	−0.205	0.436	−0.326
PC2	0.871	0.525	−0.187	0.490	−0.281
PC3	0.885	0.453	−0.118	0.425	−0.227
PC4	0.888	0.462	−0.112	0.472	−0.240
SR1	0.503	0.828	−0.208	0.694	−0.308
SR2	0.388	0.824	−0.103	0.583	−0.182
SR3	0.498	0.834	−0.185	0.571	−0.290
SR4	0.428	0.850	−0.225	0.575	−0.322
PU1	−0.187	−0.218	0.921	−0.185	0.709
PU2	−0.188	−0.185	0.912	−0.171	0.693
PU3	−0.125	−0.164	0.872	−0.125	0.628
PU4	−0.126	−0.217	0.924	−0.143	0.734
RT1	0.393	0.691	−0.164	0.830	−0.267
RT2	0.475	0.551	−0.089	0.795	−0.252
RT3	0.466	0.565	−0.127	0.830	−0.253
RT4	0.401	0.611	−0.197	0.864	−0.282
PI1	−0.295	−0.359	0.711	−0.344	0.934
PI2	−0.335	−0.317	0.669	−0.267	0.900
PI3	−0.233	−0.244	0.713	−0.263	0.904
PI4	−0.251	−0.282	0.701	−0.276	0.923

**Table A3:** Results of discriminant validity.

	Privacy Concerns (PC)	Sacrifice of Resources (SR)	Perceived Usefulness (PU)	Resistance (RT)	Participation Intention (PI)
PC	(0.880)				
SR	−0.305	(0.834)			
PU	−0.177	−0.218	(0.908)		
RT	0.520	0.732	−0.175	(0.830)	
PI	−0.305	−0.332	0.763	−0.318	(0.915)

The number in parentheses is the square root of AVE. The numbers not in the parentheses are correlations.
